# Supplementary material for: Implementation of Australia’s renewed cervical screening program: Preparedness of general practitioners and nurses
Source: PLoS One. 2020 Jan 29;15(1):e0228042. doi: 10.1371/journal.pone.0228042 (PMC6988932; doi:10.1371/journal.pone.0228042)
Supplement: S1 File — (PDF) [file pone.0228042.s001.pdf]

VCS

Implementation of the new National Cervical Screening Program in General Practice

General practitioners (GPs) play an important role in educating women about the potential benefits of participation in routine cervical screening as well as the delivery of the program. **We are conducting a 10 minute survey to understand how prepared GPs feel to undertake cervical screening under the new guidelines from 1st December 2017.** Your responses will help us improve resources and current systems to ensure GPs continue to contribute to the success of the National Cervical Screening Program.

Please answer as the questions or statements apply to you in your current practice *this week*.

<u>All information provided will be confidential and you will not be identifiable in any analysis or reporting of the survey.</u>

**1.** Your age ☐☐ years	**2.** Main practice postcode ☐☐☐☐
**3.** Your gender ☐ (**F**=female; **M**=male)	**4.** Amount of time in general practice<br>☐☐ months ☐☐ years
**5.** Your role in the practice?<br>a. General Practitioner ☐<br>b. GP registrar ☐<br>c. Nurse ☐<br>d. Other (specify) ☐ ____________	**6.** How many hours do you work at the practice per week?<br>☐☐ Hours
**7.** a. Average number of **<u>female</u>** patients you see in a week? ☐☐☐<br><br>b. How many of them are **<u>under 25 years</u>**?<br>☐☐☐	**8.** Please indicate which, if any, preventive services you routinely offer to asymptomatic sexually active female patients **<u>aged less than 25 years</u>**?<br>a. Pap testing ☐<br>b. Contraceptive services ☐<br>c. Mental Health ☐<br>d. Chlamydia testing ☐<br>e. Other STI services ☐<br>f. Immunization ☐<br>g. HEADSS screen ☐
**9.** How many Pap tests do you do in a month?<br>☐☐☐	**10.** How confident do you **<u>currently</u>** feel discussing Pap testing with eligible women?<br>a. Not at all confident ☐<br>b. Somewhat confident ☐<br>c. Confident ☐<br>d. Extremely confident ☐
**11.** How confident do you **<u>currently</u>** feel discussing Pap testing with women who are reluctant to have a Pap test?<br>a. Not at all confident ☐<br>b. Somewhat confident ☐<br>c. Confident ☐<br>d. Extremely confident ☐	**12.** What is your main source of information about the changes to the National Cervical Screening Program in Australia? (e.g. colleagues, medical media, government communication, journals etc…)<br>a. Colleagues ☐<br>b. Medical media ☐<br>c. Government communication ☐<br>d. Journals ☐<br>e. Other (specify) ____________ ☐

**13.** How **comfortable** do you feel **today** about your ability to implement the following under the new cervical screening guidelines (**PLEASE CIRCLE**)	Not at all comfortable	Fairly uncomfort able	Slightly uncomfortable	Comfortable enough	Extremely comfortable
a. Only offering routine cervical screening to women 25 years and over	1	2	3	4	5
b. Screening HPV negative women every 5 years	1	2	3	4	5
c. Collecting **only** a liquid based sample (not preparing a slide)	1	2	3	4	5
d. Referring all HPV16/18 positive women for colposcopy regardless of their cytology result	1	2	3	4	5
e. Not referring other oncogenic HPV positive (non HPV16/18) women who have low grade/negative cytology for colposcopy	1	2	3	4	5
f. Recommending self-collection to an under-screened woman who refuse a practitioner-collected cervical sample	1	2	3	4	5
g. Having to wait to offer a repeat self-collection until the woman is overdue again (7 years since last screen)	1	2	3	4	5

**14.** How **confident** do you feel **today** about your ability to implement the following (**PLEASE CIRCLE**)		Not at all confident	Not very confident	Confident enough	Very confident
a. Recommend HPV screening to a woman		1	2	3	4
b. Explain the association between HPV and cervical cancer to a woman		1	2	3	4
c. Explain a 16/18 positive HPV test to a woman		1	2	3	4
d. Explain a non 16/18 (other oncogenic) HPV positive test result to a woman		1	2	3	4
e. Explain a negative HPV test result to a woman who will be asked to return in 5 years		1	2	3	4
f. Explain to a woman aged less than 25 years why she is not eligible for routine cervical screening		1	2	3	4
g. Explain to a woman why more frequent cervical screening (i.e. every 2 years) is no longer recommended		1	2	3	4
h. Discuss the self-collection option with an eligible (under-screened) woman		1	2	3	4
i. Explain to a woman who is not eligible for self-collection why this is the case		1	2	3	4

**15.** Please indicate whether **<u>you agree or disagree</u>** with each of the following statements (**PLEASE TICK**)	Agree	Disagree	Don't know
a. I have access to educational materials to support my patients under the new program	☐	☐	☐
b. I know where to find the new guidelines (2016) for cervical screening	☐	☐	☐
c. I clearly understand which patients will be eligible for the self-collection pathway	☐	☐	☐
d. Self-collection is a reliable test	☐	☐	☐
e. I understand in what way the reminder and recall systems in my practice will need to change under the new program	☐	☐	☐
f. Staff in my practice can easily access materials in the work place that support them in implementing the new program	☐	☐	☐
g. I have patient information about the new screening program in my waiting area	☐	☐	☐
h. I understand how the national cancer screening register will support the new program	☐	☐	☐
i. I know how I will obtain information about my patients from the national cancer screening register	☐	☐	☐
j. I know who to contact if I have questions about screening results and recommendations for my patients	☐	☐	☐
k. I trust the provider of the national cancer screening register (Telstra Health) with my patient's data	☐	☐	☐

16. In your opinion what are the **<u>key barriers</u>** to implementing the new cervical screening program in General Practice

____

____

____

____

17. Briefly outline how you think you might overcome these barriers in your practice.

____

____

____

____

18. Do you have any other comments or concerns about the new cervical screening program you would like to share?

____

____

____

____

We would like to thank you for your time in filling out this questionnaire.
