# Supplementary material for: Implementation of Australia’s renewed cervical screening program: Preparedness of general practitioners and nurses
Source: PLoS One. 2020 Jan 29;15(1):e0228042. doi: 10.1371/journal.pone.0228042 (PMC6988932; doi:10.1371/journal.pone.0228042)
Supplement: S2 File — (DOCX) [file pone.0228042.s002.docx]

**Supplementary file S2: Major themes and subthemes from open-ended responses.**

| Major themes and subthemes around barriers to implementation of the renewed program | Pre-renewal responses (n=127) | Post-renewal responses  (n=125) |
| --- | --- | --- |
|  | n1 | n2 |
| Concerns regarding delayed screening interval   - Patient’s fear of cervical cancer being diagnosed - Patient accustomed to the 2 year interval and resistant to change - Practitioner’s concerns about loss to patient follow-up - Practitioner’s concerns around reduced screening opportunities for other diseases | 29 | 5 |
| Concerns regarding delayed starting age for cervical screening   - Practitioner’s concerns around cervical cancer being missed in young women and sexually active young women - Explaining and getting patients to understand and accept this change - Practitioner’s concern that women may get busy by 25 | 12 | 4 |
| Lack of patient’s understanding and education around the changes   - Lack of understanding of the changes in general - Lack of knowledge about HPV and the test - Lack of awareness around self-collection - Scant or incorrect information in the media | 48 | 40 |
| Lack of providers understanding and education around the changes   - Lack of practitioners’ understanding and awareness around the changes - Lack of other clinic staff understanding and awareness around the changes - Limited information and education materials to provide to patients - More information around what to do in specific situations | 28 | 37 |
| Register and system related issues   - Delay in implementing the register - Integration with the register - Concerns around effective reminder systems | 14 | 11 |
| Concerns around self-collection   - Eligibility around self-collection - Waiting time around self-collection - Poor publicity of self-collection - Patient lacking understanding that testing positive would mean they need to come back to the clinic | 4 | 3 |
| Others   - Changing what people are familiar with takes time - More time to explain the changes to people initially - Getting patients to clinic | 8 | 28 |
